# Supervised Multi-Layer Conditional Variational Auto-Encoder for Process Modeling and Soft Sensor

**DOI:** 10.3390/s23229175

**Published:** 2023-11-14

**Authors:** Xiaochu Tang, Jiawei Yan, Yuan Li

**Affiliations:** 1 School of Automation, Shenyang Aerospace University, Shenyang 110136, China; yanjiawei@stu.sau.edu.cn; 2 College of Information Engineering, Shenyang University of Chemical Technology, Shenyang 110142, China; li-yuan@mail.tsinghua.edu.cn

**Keywords:** soft sensor, deep learning, supervised model, variational auto-encoder

## Abstract

Variational auto-encoders (VAE) have been widely used in process modeling due to the ability of deep feature extraction and noise robustness. However, the construction of a supervised VAE model still faces huge challenges. The data generated by the existing supervised VAE models are unstable and uncontrollable due to random resampling in the latent subspace, meaning the performance of prediction is greatly weakened. In this paper, a new multi-layer conditional variational auto-encoder (M-CVAE) is constructed by injecting label information into the latent subspace to control the output data generated towards the direction of the actual value. Furthermore, the label information is also used as the input with process variables in order to strengthen the correlation between input and output. Finally, a neural network layer is embedded in the encoder of the model to achieve online quality prediction. The superiority and effectiveness of the proposed method are demonstrated by two real industrial process cases that are compared with other methods.

## 1. Introduction

It is important for effective quality control and process monitoring to obtain quality or key variables accurately and timely in industrial processes. However, these key variables are often difficult to measure directly, expensive to acquire, or delayed in analysis, which restricts the development of quality control. To make quality or key variables available online, the data-driven soft sensor has been widely applied to build prediction models between easy-to-measure process variables and hard-to-measure quality or key variables [1,2,3].

Traditional modeling methods, such as partial least squares (PLS) and principal component regression (PCR), have been widely studied and applied to industrial processes [4,5]. Although these methods can achieve online quality prediction, the prediction accuracy is difficult to meet the accuracy requirement for complex nonlinear processes as the result of nonlinearity between the process variables is not considered in the modeling process. In the past decades, some nonlinear modeling methods have been put forward to improve the performance of data-driven soft sensors. For example, some shallow learning methods such as support vector regression, Gaussian process regression, and neural networks have been introduced for nonlinear soft sensors [6,7,8]. In these methods, nonlinear information can be definitely explicitly considered. However, these shallow networks seem to have inadequate representation capabilities for complex nonlinear processes.

Recently, due to the superiority of nonlinear feature extraction, deep learning has been introduced for process monitoring and soft sensors [9,10,11,12]. Shang et al. proposed a deep belief network (DBN) model for sensors to fully extract nonlinear process characteristics [13]. Liu et al. designed a multilayer DBN to represent the nonlinear relationship between the flame images and the outlet oxygen content [14]. Yan et al. combined a de-noising auto-encoder with a neural network to establish a soft sensor model [15]. Yuan et al. extracted the quality-relevant nonlinear feature by constructing a variable-wise weighted stacked auto-encoder [16,17]. Yao and Ge developed a deep soft sensor model based on a hierarchical extreme learning machine [18]. Wang et al. integrated a long short-term memory with a stacked auto-encoder to achieve quality prediction in a batch process [19]. Feng et al. constructed a dual attention-based encoder-decoder based on the long short-term memory network [20]. In these methods, nonlinear features can be well described and represented. However, these methods lack the description abilities of data measurement noise and process uncertainty. To date, the variational auto-encoder (VAE) as a deep generative model has attracted increasing attention and has also been successfully applied to process modeling [21].

Jiang et al. proposed a variational deep embedding method to improve the data generative process of the VAE method [22]. Zhao et al. proposed a generative method by embedding truncated GMM in VAE to capture the multi-modal representation of data including outlier samples [23]. Dilokthanakul et al. used GMM as a prior distribution to substitute for traditional prior distribution in VAE [24]. Liu et al. obtained a new approximate posterior distribution of VAE to match the true posterior distribution [25]. However, VAE is an unsupervised deep generative model so it cannot be directly applied to soft sensor modeling. While existing process modeling methods based on VAE mainly focus on unsupervised modeling for fault detection and diagnosis, only a few supervised VAE models are developed for soft sensors in industrial processes. Shen et al. proposed a supervised nonlinear probabilistic latent variable regression model based on VAE for soft sensors [26,27]. In this method, nonlinear and dynamic features can be extracted simultaneously. However, VAE only is used to extract nonlinear characteristics between input variables, and the construction of the regression model is actually based on the additional neural networks. Guo et al. developed a Gaussian mixture variational auto-encoder to deal with the problem of multimode soft sensors [28,29]. Similarly, the regression model is built based on a mixture of the probabilistic principal component regression (MPPCR) model, rather than a supervised VAE framework. Xie et al. extended the unsupervised VAE to the supervised VAE by combining the encoder of unsupervised VAE with the decoder of supervised VAE [30]. It may construct a supervised VAE framework for soft sensors in a real sense. However, this method depends on the assumption that the distribution of two different latent variables’ spaces are approximately the same. Whether the conditions are strictly met may affect the accuracy of the model.

It is a more critical problem, no matter what unsupervised VAE or supervised VAE, that the generated output is random and uncontrollable only according to the distribution. Therefore, it is critical for a variational auto-encoder to constrain and control data generation. Based on such an idea, a multi-layer conditional variational auto-encoder (M-CVAE) is proposed in order to improve stability and controllability without additional output networks. First, a CVAE model is constructed by inputting the label information to the input of the decoder with the latent variables [31,32]. In this way, the output data can be generated towards the target direction according to the label information instead of randomly. Furthermore, the key label information is introduced to the input of the CVAE model in order to strengthen the correlation between the input and output. Finally, a multi-label layers network is added to the CVAE framework to achieve online prediction.

The rest of this article is structured as follows. The traditional VAE model is introduced in Section 2. The proposed M-CVAE and two industrial cases that are given to demonstrate the effectiveness of the proposed method are illustrated in Section 3. Finally, the conclusions are made.

## 2. Variational Auto-Encoder

**Figure 1:**
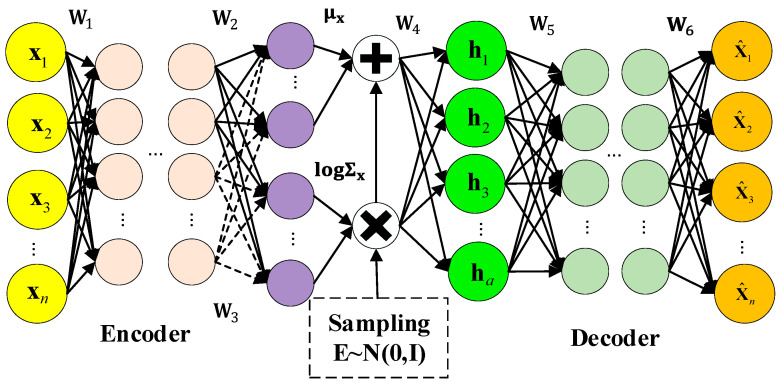
The model structure of VAE.

A variational auto-encoder (VAE) is an unsupervised deep generative model. In VAE, Bayesian probability is introduced to the neural network so that it can learn complex data distribution from a probabilistic latent variable space. The model structure of VAE is shown in Figure 1. The encoder and decoder are included in VAE. In the encoder, input data x are mapped to the latent space so that the latent variables h are obtained. The latent variables h are input to the decoder to obtain the output $\hat{x}$.

For VAE, instead of the joint distribution of input variables, the marginal likelihood can be calculated as:

(1) $$p_{\theta }\left(x\right)=\int_{h}p_{\theta }(h)p_{\theta }\left(x|h\right)dh$$

where $p_{\theta }\left(h\right)$ is the prior of the latent variables, which follows the Gaussian distribution with parameter θ. Because h and θ are unknown, the marginal likelihood is intractable. The latent variable h is obtained from the input data x and $p_{\theta }\left(h\right)$ can be substituted for $p_{\theta }(h|x)$. The true posterior $p_{\theta }(h|x)$ is given by

(2) $$p_{\theta }\left(h|x\right)=p_{\theta }\left(h\right)p_{\theta }\left(x|h\right)/p_{\theta }\left(x\right)$$

here, the method of variational inference is introduced. Here, $q_{\phi }\left(h|x\right)$ follows Gaussian distribution with parameter ϕ, which is used to approximate true posterior distribution. Furthermore, the marginal log-likelihood can be written as:

(3) $$\begin{array}{l} \log p_{\theta }\left(x\right)=\int_{h}q_{\phi }\left(h|x\right)\log p_{\theta }\left(x\right)dh\\ \;\;\;\;\;=\int_{h}q_{\phi }\left(h|x\right)\log p_{\theta }\left(x,h\right)/p_{\theta }\left(h|x\right)dh\\ \;\;\;\;\;=\int_{h}q_{\phi }\left(h|x\right)\log\left(\frac{p_{\theta }\left(x,h\right)}{q_{\phi }\left(h|x\right)}\cdot \frac{q_{\phi }\left(h|x\right)}{p_{\theta }\left(h|x\right)}\right)dh\\ \;\;\;\;\;=\int_{h}q_{\phi }\left(h|x\right)\log\frac{p_{\theta }\left(x,h\right)}{q_{\phi }\left(h|x\right)}dh+\int_{h}q_{\phi }\left(h|x\right)\log\frac{q_{\phi }\left(h|x\right)}{p_{\theta }\left(h|x\right)}dh\\ \;\;\;\;\;=\mathrm{ELBO}+D_{\mathrm{KL}}\left(q_{\phi }\left(h|x\right),p_{\theta }\left(h|x\right)\right) \end{array}$$

where the marginal log-likelihood is divided into two parts: evidence lower bound (ELBO) and KL divergence between $q_{\phi }(h|x)$ and $p_{\theta }(h|x)$. The ELBO can be further written as:

(4) $$\begin{array}{l} \mathrm{ELBO}=\int_{h}q_{\phi }\left(h|x\right)\log\left(p_{\theta }\left(x,h\right)/q_{\phi }\left(h|x\right)\right)dh\\ \;\;\;=\int_{h}q_{\phi }\left(h|x\right)\log p_{\theta }\left(x,h\right)p\left(h\right)/q_{\phi }\left(h|x\right)dh\\ \;\;\;=\int_{h}q_{\phi }\left(h|x\right)\log p\left(h\right)/q_{\phi }\left(h|x\right)dh+\int_{h}q_{\phi }\left(h|x\right)\log p_{\theta }\left(x|h\right)dh\\ \;\;\;=-D_{\mathrm{KL}}\left(q_{\phi }\left(h|x\right),p_{\theta }\left(h|x\right)\right)+\;E_{q_{\phi }}\left[\log p_{\theta }\left(x|h\right)\right] \end{array}$$

where $D_{\mathrm{KL}}\left(q_{\phi }\left(h|x\right),p_{\theta }\left(h|x\right)\right)$ is the divergence of $p_{\theta }\left(h|x\right)$ and $q_{\phi }\left(h|x\right)$. $\;E_{q_{\phi }}\left[\log p_{\theta }\left(x|h\right)\right]$ is the reconstructed error of $\log p_{\theta }\left(x|h\right)$. Maximizing the marginal probability function can be converted to maximizing the evidence lower bound. Thus, maximizing ELBO can be written as:

(5) $$\underset{\theta ,\phi }{\max}\mathrm{ELBO}=\underset{\theta ,\phi }{\max}\left\{E_{q_{\phi }}\left[\log p_{\theta }\left(x|h\right)\right]-D_{\mathrm{KL}}\left(q_{\phi }\left(h|x\right),p_{\theta }\left(h|x\right)\right)\right\}$$

Prior $p_{\theta }(x)$ follows Gaussian distribution with zero mean and variance 1, and the approximate posterior $q_{\phi }(h|x)$ is assumed to follow a multivariate Gaussian with mean $\mu_{x}$ and variance $\Sigma_{x}$. The KL divergence is:

(6) $$\begin{array}{l} D_{\mathrm{KL}}\left(q_{\phi }\left(h|x\right),p_{\theta }\left(h|x\right)\right)=\int_{h}N\left(\mu_{x},\Sigma_{x}\right)\log N\left(\mu_{x},\Sigma_{x}\right)/N\left(0,I\right)dh\\ \;\;\;\;\;\;\;\;\;\;\;\;\;=-\frac{1}{2}\sum_{t=1}^{T}\left(1+\log{\left(\Sigma_{x}^{t}\right)}^{2}-{\left(\mu_{x}^{t}\right)}^{2}-{\left(\Sigma_{x}^{t}\right)}^{2}\right) \end{array}$$

where $\mu_{x}^{t}$ and $\Sigma_{x}^{t}$ denote the mean and variance of the sample at t-th time, t=1,2,⋯,T. The loss function of the VAE can be simplified as:

(7) $$\begin{array}{l} J(\theta ,\phi ;x)=-D_{\mathrm{KL}}\left(q_{\phi }\left(h|x\right),p_{\theta }\left(h|x\right)\right)+E_{q_{\phi }}[\log p_{\theta }\left(x|h\right)]\\ \;\;\;\;=\frac{1}{2}\sum_{t=1}^{T}\left(1+\log{\left(\Sigma_{x}^{t}\right)}^{2}-{\left(\mu_{x}^{t}\right)}^{2}-{\left(\Sigma_{x}^{t}\right)}^{2}\right)+\frac{1}{T}\sum_{t=1}^{T}\log p_{\theta }\left(x|h^{\left(t\right)}\right) \end{array}$$

where T is the sampling number and $h^{\left(t\right)}$ denotes the latent variable of the t-th sample.

## 3. Methodology

In this section, the proposed multi-layer conditional variational auto-encoder (M-CVAE) is presented. Firstly, the structure of the M-CVAE model is described. The derivation of the M-CVAE algorithm is further given. Finally, the procedures of the soft sensor based on the M-CVAE model are presented.

### 3.1. Supervised Multi-Layer Conditional Variational Auto-Encoder

**Figure 2:**
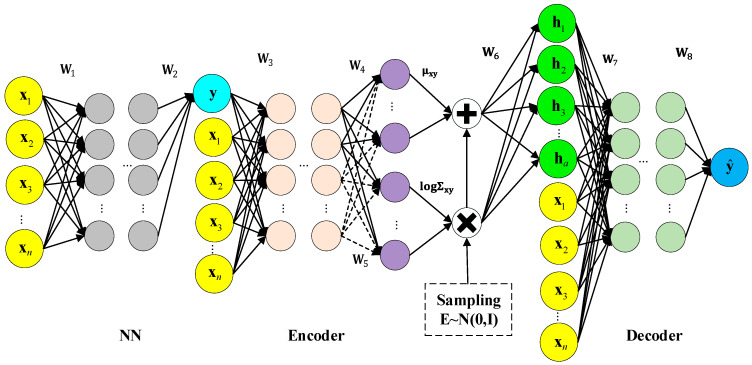
The model structure of M-CVAE.

VAE provides an unsupervised modeling framework. However, it cannot be applied to soft sensor modeling between process variables and quality variables. A supervised M-CVAE structure is constructed in this section, as shown in Figure 2. In M-CVAE, a conditional model structure is constructed based on the basic VAE framework. The label x as a condition is added to the input of the encoder and decoder of VAE. The input of the encoder becomes a concatenation of the original data y and label information x, while the output remains unchanged. The input of the decoder becomes a concatenation of normal distribution sampling corresponding to latent variable h and label information, while the output remains unchanged. In this way, the label of the encoder can constrain the resampling range in specified label areas, rather than the entire normal distribution. The label of the decoder can control the generation of data according to the specified labels or conditions. Compared with basic VAE, in such a condition structure, data generation is still based on the probability distribution of latent variable space with noise. However, the data generation of the CVAE model is no longer entirely random, but becomes targeted by introducing conditions so that the prediction results become controllable rather than stochastic.

In order to achieve online quality prediction, a multi-layer CVAE model is further constructed by adding a neural network layer to the CVAE model. The effect of the neural network is that it generates the initial predicted y that is used as the input of CVAE. Then, the initial prediction y with the corresponding label is injected into the CVAE model to make the final prediction closer to the actual value. The neural network layer has multiple hidden layers, which aim to match the input data y of CVAE and labels, to ensure the robustness and generalization ability of the CVAE model, and generate the initial prediction y as the input of CVAE, so that the final prediction value can be generated after passing through the conditional encoder and conditional decoder with x as the condition. Based on the neural network in front of CVAE, on the one hand, the input data y of CVAE and labels can be matched, and on the other hand, online prediction can be achieved.

Similar to the VAE model, to train the model, it is necessary to obtain the log-likelihood function, and the log-likelihood of M-CVAE can be written as follows:

(8) $$\begin{array}{l} \log p_{\theta }\left(y|x\right)=\int_{h}q_{\phi }\left(h|x,y\right)dh\cdot \log q_{\phi }\left(y|x\right)\\ \;\;\;\;\;=\int_{h}q_{\phi }\left(h|x,y\right)\log p_{\theta }\left(y|x\right)dh\\ \;\;\;\;\;=\int_{h}q_{\phi }\left(h|x,y\right)\log\left(p_{\theta }\left(h,x,y\right)/p_{\theta }\left(h|x,y\right)p_{\theta }\left(x\right)\right)dh\\ \;\;\;\;\;=\int_{h}q_{\phi }\left(h|x,y\right)\log\left(\frac{p_{\theta }\left(h,x,y\right)}{q_{\phi }\left(h|x,y\right)p_{\theta }\left(x\right)}\cdot \frac{q_{\phi }\left(h|x,y\right)}{p_{\theta }\left(h|x,y\right)}\right)dh\\ \;\;\;\;\;=\int_{h}q_{\phi }\left(h|x,y\right)\log\left(p_{\theta }\left(h,x,y\right)/\left(q_{\phi }\left(h|x,y\right)p_{\theta }\left(x\right)\right)\right)dh+\int_{h}q_{\phi }\left(h|x,y\right)\log\left(q_{\phi }\left(h|x,y\right)/p_{\theta }\left(h|x,y\right)\right)dh\\ \;\;\;\;\;=\mathrm{ELBO}+D_{\mathrm{KL}}\left(q_{\phi }\left(h|x,y\right),p_{\theta }\left(h|x,y\right)\right) \end{array}$$

where it can be estimated by the neural network of S-DCVAE, x is a process variable and also serves as a condition for the model, h is the latent variable, and $q_{\phi }\left(y|x\right)$ is used to approximate $p_{\theta }\left(y|x\right)$. ELBO was used as the objective function in variational inference. The log-likelihood function can be indirectly maximized by maximizing ELBO. ELBO can be further written as:

(9) $$\begin{array}{l} \mathrm{ELBO}=\int_{h}q_{\phi }\left(h|x,y\right)\log\left(p_{\theta }\left(h,x,y\right)/q_{\phi }\left(h|x,y\right)\cdot p_{\theta }\left(x\right)\right)dh\\ \;\;\;\;\;\;\;=\int_{h}q_{\phi }\left(h|x,y\right)\log\frac{p_{\theta }\left(y|x,h\right)p_{\theta }\left(h|x\right)p_{\theta }\left(x\right)}{q_{\phi }\left(h|x,y\right)p_{\theta }\left(x\right)}dh\\ \;\;\;\;\;\;\;=\int_{h}q_{\phi }\left(h|x,y\right)\log\left(p_{\theta }\left(h|x\right)/q_{\phi }\left(h|x,y\right)\right)dh+\int_{h}q_{\phi }\left(h|x,y\right)\log p_{\theta }\left(y|x,h\right)dh\\ \;\;\;\;\;\;\;=-D_{\mathrm{KL}}\left(q_{\phi }\left(h|x,y\right),p_{\theta }\left(h|x\right)\right)+E_{q_{\phi }}\left[\log p_{\theta }\left(y|x,h\right)\right]\; \end{array}$$

Furthermore, maximizing ELBO can be written as:

(10) $$\underset{\theta ,\phi }{\max}\mathrm{ELBO}=\underset{\theta ,\phi }{\max}\left\{E_{q_{\phi }}\left[\log p_{\theta }\left(y|x,h\right)\right]-D_{\mathrm{KL}}\left(q_{\phi }\left(h|x,y\right),p_{\theta }\left(h|x\right)\right)\right\}$$

where the approximate posterior $q_{\phi }\left(h|x,y\right)$ is assumed to be a multivariate Gaussian distribution with mean $\mu_{xy}$ and variance $\Sigma_{xy}$. The KL divergence can be given as follows:

(11) $$\begin{array}{l} D_{\mathrm{KL}}\left(q_{\phi }\left(h|x,y\right),p_{\theta }\left(h|x\right)\right)=\int_{h}N\left(\mu_{xy},\Sigma_{xy}\right)\log\left(N\left(\mu_{xy},\Sigma_{xy}\right)/N\left(0,I\right)\right)dh\\ \;\;\;\;\;\;\;\;\;\;\;\;=-\frac{1}{2}\sum_{t=1}^{T}\left(1+\log{\left(\Sigma_{xy}^{t}\right)}^{2}-{\left(\mu_{xy}^{t}\right)}^{2}-{\left(\Sigma_{xy}^{t}\right)}^{2}\right) \end{array}$$

Therefore, the loss function of the supervised M-CVAE can be written as follows:

(12) $$\begin{array}{l} J(\theta ,\phi ;x,y)=-D_{\mathrm{KL}}\left(q_{\phi }\left(h|x,y\right),p_{\theta }\left(h|x\right)\right)+\;E_{q_{\phi }}\left[\log p_{\theta }\left(y|x,h\right)\right]\;\\ \;\;\;\;\;\;\;\;\;\;\;=\frac{1}{2}\sum_{t=1}^{T}\left(1+\log{\left(\Sigma_{xy}^{t}\right)}^{2}-{\left(\mu_{xy}^{t}\right)}^{2}-{\left(\Sigma_{xy}^{t}\right)}^{2}\right)+\frac{1}{T}\sum_{t=1}^{T}\log p_{\theta }\left(y|x,h^{\left(t\right)}\right) \end{array}$$

where T is the number of samples and $h^{\left(t\right)}=\mu_{xy}^{\left(t\right)}+\Sigma_{xy}^{\left(t\right)}\times \varepsilon ,$ ε∼N(0,I) is obtained by reparameterization.

### 3.2. Soft Sensor Based on M-CVAE

In this section, a detailed derivation of the M-CVAE model has been illustrated and M-CVAE is applied for an online soft sensor. A series of online samples, denoted as $x_{new}^{t}$, have been obtained. The predicted variable $y_{new}^{t}$ can be given as follows:

(13) $$\begin{array}{l} y_{new}^{t}=f\left(x_{new}^{t}\right)\\ h_{new}^{t}=\mu_{{}_{xy_{new}}}^{t}+\Sigma_{{}_{xy_{new}}}^{t}\times \varepsilon ,\;\varepsilon ∼N(0,I)\\ {\hat{y}}_{new}^{t}=g(h_{new}^{t}) \end{array}$$

where $y_{new}^{t}$ is the quality value obtained from the neural network, and f(∗) and g(∗) are the nonlinear functions corresponding to the neural network layer and CVAE, respectively. $h_{new}^{t},\;\mu_{xynew}^{t},\;\Sigma_{xynew}^{t}$ are the latent variable, mean, and variance of the online sample $x_{new}^{t}$. The main procedures for the M-CVAE are summarized as follows:

1. Collect input data and output data for the training set.
2. Determine the process variables for and standardize the training set.
3. Initialize M-CVAE network parameters θ, ϕ.
4. Train the M-CVAE model for output prediction.
5. Choose a different number of the latent variables for the M-CVAE model. Repeat step 4 to determine the optimal number of latent variables.
6. Collect the test data and standardize the test data set.
7. Predict the quality variable ${\hat{y}}_{new}^{t}$.

**Figure 3:**
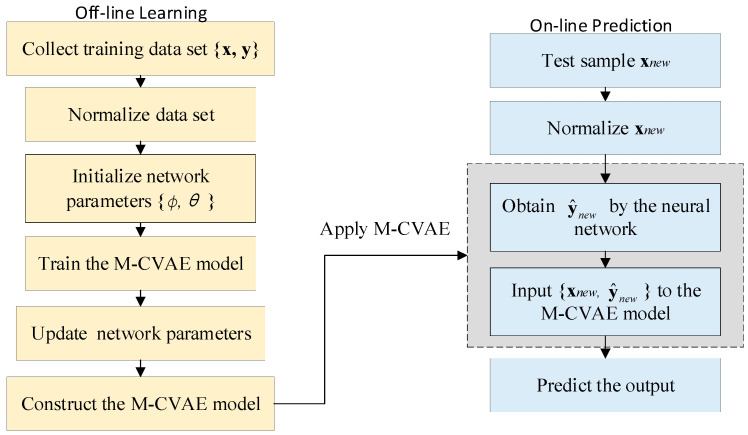
The flowchart of M-CVAE.

The flowchart of M-CVAE is provided in Figure 3.

### 3.3. Case Studies

In this section, the M-CVAE model is applied to two real industrial cases for soft sensors. To evaluate the performance of the proposed M-CVAE, three indexes, the mean absolute error (MAE), the root mean square error (RMSE), and the coefficient of determination index R^2^, are calculated comparing the results with other regression models.

#### 3.3.1. Debutanizer Column

**Figure 4:**
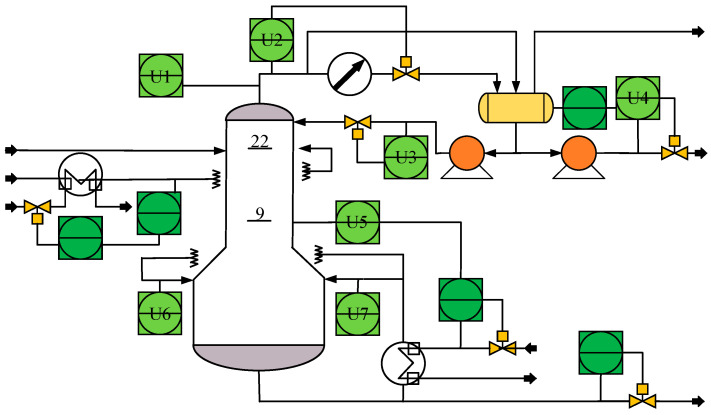
The flowchart of the debutanizer column.

The debutanizer column is an important part of the refinery process in petroleum production processes, which can separate propane and butane from the naphtha stream [33]. The process flowchart is shown in Figure 4. Due to the butane content at the bottom of the debutanizer column being very low, the measurement of butane concentration is difficult and there is usually a great delay. Therefore, it is valuable to introduce the soft senor for butane concentration.

**Figure 5:**
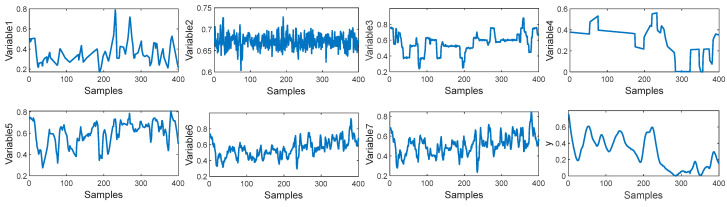
The trend distributions of input variables and output variables.

In this paper, a total of 2394 samples have been collected from the debutanizer column. The first 1900 samples are used for the training set and the last 400 samples are used for the test set. Seven process variables are selected as the input of the M-CVAE model to predict the butane concentration and a detailed description of the seven process variables and the output of the predicted variable is shown in Table 1. The trend of input variables and output variables is shown in Figure 5. It can be seen that the input of these variables has obvious fluctuations, which indicates that there is significant nonlinearity in such a process. In order to demonstrate the nonlinearity between the input variables and output variables, the degree of repeatability (DR) and the differential degree of repeatability (DDR) are introduced [34]. The DR and DDR can reflect the similarity and difference of the correlation of the sample blocks.

**Figure 6:**
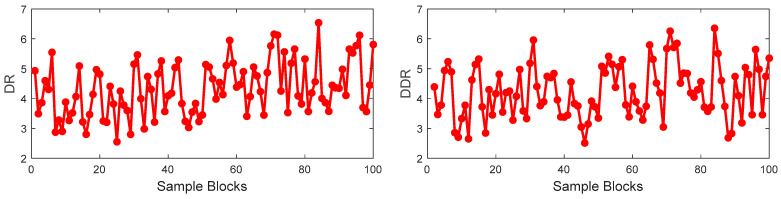
The trend of correlation between input variables and output variable of the prosses.

Firstly, the training set is divided into 100 blocks with 19 samples in each block. The DR of each block and the DDR of two adjacent blocks are calculated as shown in Figure 6. In Figure 6, the values of the DR and DDR have random change among the sample blocks, which indicates that the correlation between input and output is not consistent throughout the whole process. It is illustrated that the nonlinearity between the process variables and output variable is obvious.

Based on the training data set, the M-CVAE model is built. For comparison, the other soft sensor models including PLS, SVR, DVAE, and Supervised NDS models are also built. Here, DVAE is supervised VAE proposed in the paper [30]. Supervised NDS is a supervised nonlinear dynamic model composed of VAE, a neural network and dynamic system, which is proposed in the paper [27].

**Figure 7:**
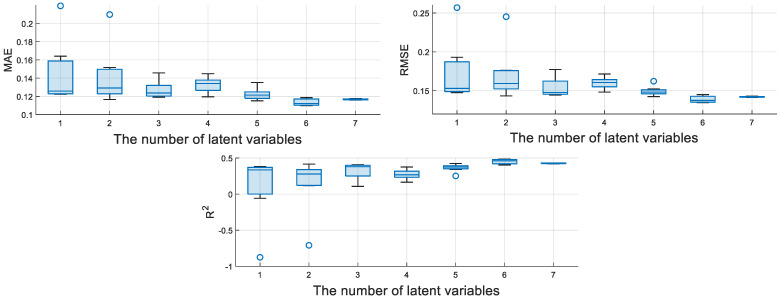
The evaluation indices versus different numbers of latent variables.

The encoder of M-CVAE consists of three convolutional layers and a fully connected layer, and the decoder consists of three deconvolution layers and a fully connected layer. The number of convolution kernels is 32, the size of the convolution kernel is 3 × 3, and the activation function is the Relu function. The number of latent variables is a key parameter, which affects the performance of the model to a certain extent. Therefore, the average evaluation indices of MAE, RMSE, and R^2^ for the M-CVAE model, which are based on the seven test experiments, are shown in Figure 7. It can show the fluctuation degree of MAE, RMSE, and R^2^ with different numbers of latent variables. That is to say, the smaller the rectangular area, the more stable the performance of the model.

Therefore, it can be seen that the M-CVAE model has the most stable performance when the number of latent variables is 7. While the number of latent variables is 6, more optimal values of MAE, RMSE, and R^2^ can be obtained. Also, the stable performance is quite excellent. Furthermore, the optimal values of MAE, RMSE, and R^2^ corresponding to different numbers of latent variables are given in Table 2. From Table 2, it can be seen that the optimal values of MAE, RMSE, and R^2^ are obtained when the number of latent variables is 6. Finally, the number of latent variables is determined as 6 for the M-CVAE model from a comprehensive point. Meanwhile, the optimal number of latent variables is also selected for other regression models.

**Figure 8:**
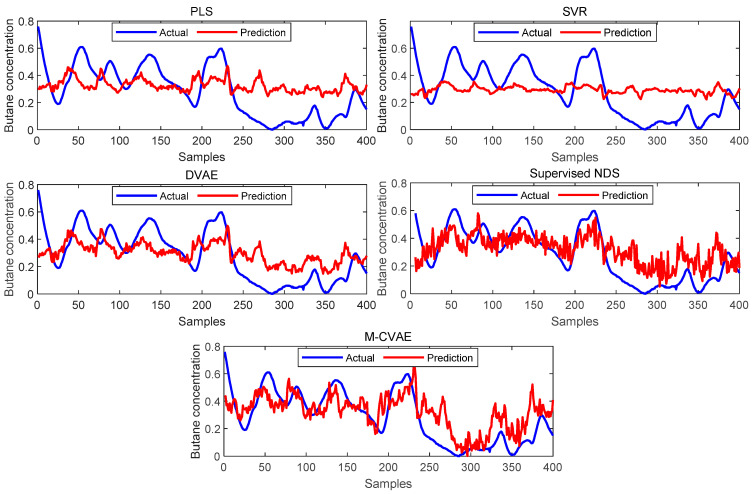
The prediction results of PLS, SVR, DVAE, Supervised NDS, and M-CVAE.

**Figure 9:**
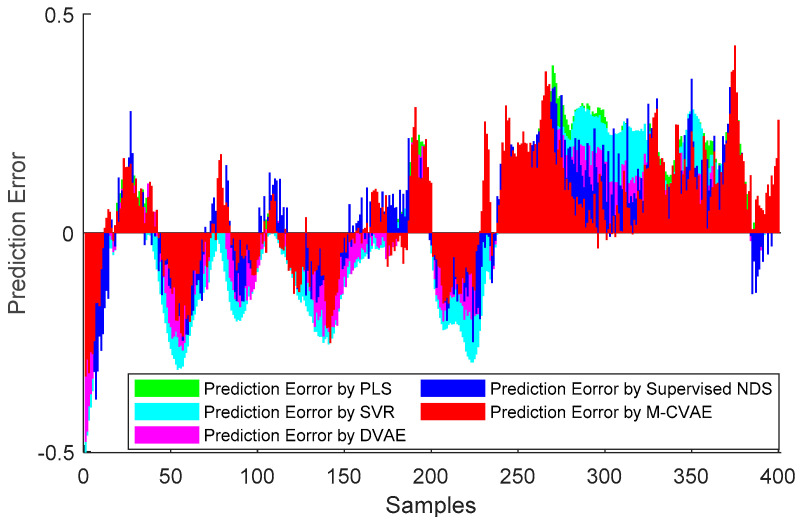
The prediction errors of PLS, SVR, DVAE, Supervised NDS, and M−CVAE.

The prediction results of PLS, SVR, DVAE, Supervised NDS, and the proposed M-CVAE method are shown in Figure 8. From Figure 8, it is not difficult to see that the predicted butane concentration based on the M-CVAE method is closest to the actual value in most of the process. It also can be seen that neither PLS nor SVR can fit the actual values well. The reason is that traditional machine learning methods are based on shallow learning, which cannot deeply mine complex nonlinearity in data. The M-CVAE has a deep generative model composed of multi-layer networks, which can well mine the nonlinear characteristics of data. With DVAE and Supervised NDS as deep learning methods, there is a better fluctuation trend of tracking the actual value. However, the fitting degree of these two methods has declined with obvious fluctuation. This is because the data generation based on both the DVAE and Supervised NDS model is similar to the traditional VAE model. While data generation is based on VAE, no constraints are introduced to the model, and as a result, the resampling can be carried out in the whole latent space. This can cause instability and uncontrollability issues in data generation. It is because as the encoding area expands, the resampling range also expands due to the noise introduced in the VAE framework. This may lead to strengthen the randomness and uncertainty of resampling, increase the sampling probability far from the original data code area, and decrease the probability of the original code area. It means that the constraint of the original data to resampling is reduced, which to some extent causes the uncontrollability and randomness of data generation. As a result, the generated data (estimated original data) output from the decoder are not as close as possible to the original data. Compared with DVAE and Supervised NDS, the proposed M-CVAE method has better traceability even at obvious fluctuation, such as samples 80th–130th, 180th–230th, and 255th–350th. The reason why M-CVAE can display the superior performance of prediction is that both process variables and butane concentration can be considered in the M-CVAE model instead of just considering process variables in DVAE and Supervised NDS. In this way, nonlinearity between process variables and butane concentration can be well extracted so that it is quite helpful for the regression model to improve the performance of prediction. It is more important that M-CVAE can control the predicted value close to the actual value by a condition instead of outputting the predicted value only according to a probability distribution, which can improve the controllability and stability of the model. In addition, labels or conditions input to the encoder and decoder are changeable with the input data. That is to say, the input data are always input to the encoder and decoder together with the matching label. Therefore, the dynamic label always can specify generated data as close as possible to the current input sample. This ensures the generalization ability and robustness of the model. Furthermore, the error prediction of all the methods is shown in Figure 9 for comparison. From Figure 9, it can be seen that the proposed M-CVAE method has the smallest error in most of the process. The detailed MAE, RMSE, and R^2^ are given in Table 3. From Table 3, we can see that M-CVAE has the lowest MAE and RMSE and the highest R^2^ among all the methods. Therefore, it can be demonstrated that the soft sensor based on M-CVAE has optimal performance.

#### 3.3.2. CO_2_ Absorption Column

**Figure 10:**
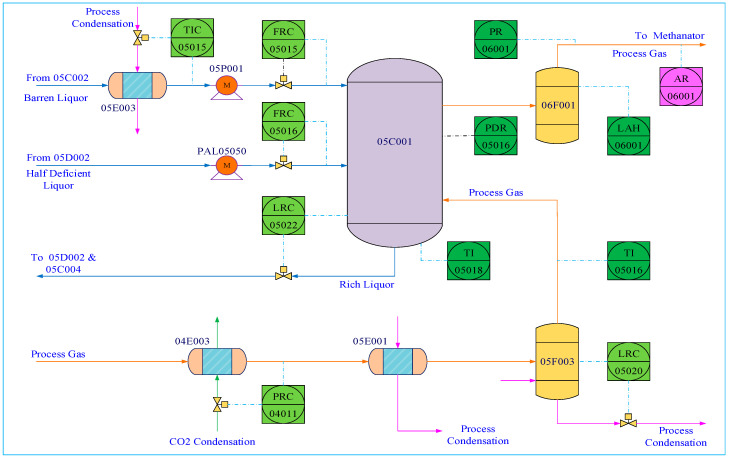
Flowchart of the CO_2_ absorption column.

The Ammonia synthesis process is a common industrial process for producing NH_3_ used as the basic material for Urea synthesis. In this process, NH_3_ is produced as well as CO_2_ so CO_2_ should be further separated. Therefore, the CO_2_ absorption column is an important unit in Ammonia synthesis for CO_2_ separation, and CO_2_ content is a key variable for quality control. The flowchart of the CO_2_ absorption column is given in Figure 10.

**Figure 11:**
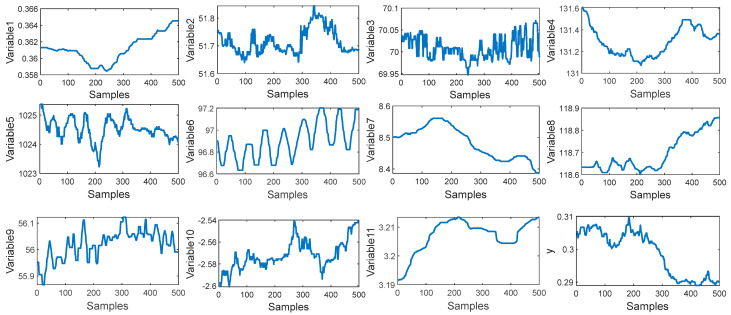
The trend distributions of input variables and output variables.

**Figure 12:**
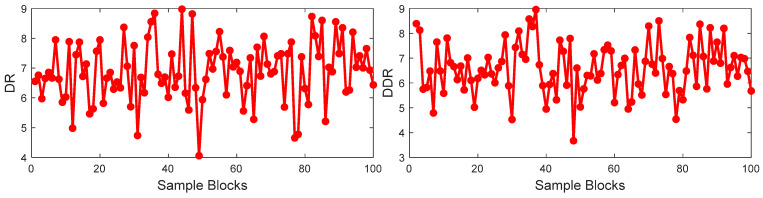
The trend of correlation between input variables and output variable.

In this paper, 11 process variables are selected for CO_2_ content prediction, which are listed in Table 4. A total of 30,000 samples are collected, in which the first 2000 samples are used as the training data set and the last 500 samples are used as the test data set. The trend of input variables and output variables is shown in Figure 11. It can be seen that the input variables and output variable have obvious fluctuation. It can be inferred that this process has a strong nonlinearity. To illustrate the nonlinearity between the input and output, the training set is divided into 100 blocks with 40 samples in each block. The DR of each block and the DDR of two adjacent blocks are shown in Figure 12. In Figure 12, the values of DR and DDR have a significant fluctuation, which illustrates that the nonlinearity between the process variables and output variable is obvious.

Based on the training data set, the model is built. The soft sensor model based on the proposed M-CVAE, PLS, SVR, DVAE, and Supervised NDS model is built. The encoder of M-CVAE consists of five convolutional layers and a fully connected layer, and the decoder consists of five deconvolution layers and a fully connected layer. The number of convolution kernels is 32, the size of the convolution kernel is 3 × 3, and the activation function is the Relu function. Similarly, the number of latent variables for the M-CVAE is determined based on the average evaluation indices of MAE, RMSE, and R^2^. The average evaluation index based on 11 test experiments is listed in Table 5. From Table 5, it can be seen that the optimal values of MAE, RMSE, and R^2^ are obtained when the number of latent variables is 11.

**Figure 13:**
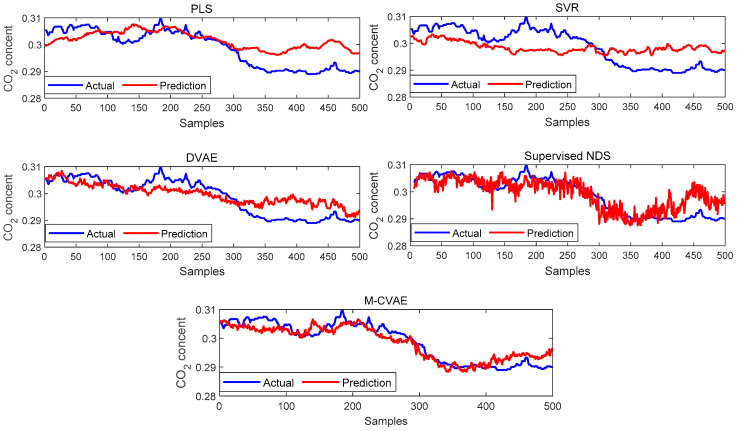
The prediction results of each model.

**Figure 14:**
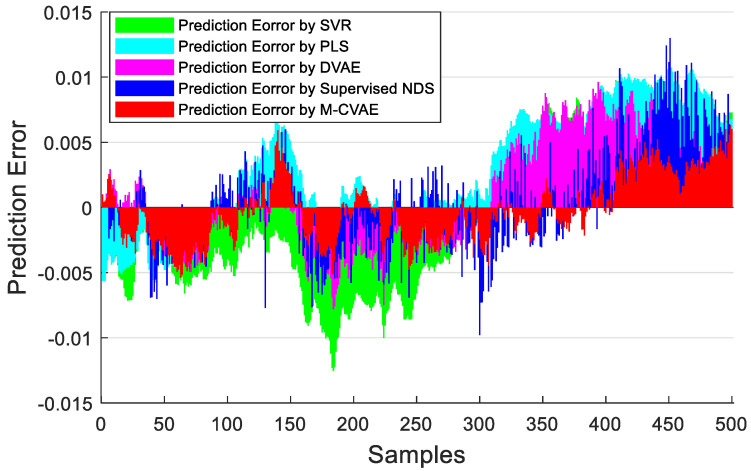
The prediction errors of each model.

The prediction results of PLS, SVR, DVAE, Supervised NDS, and M-CVAE are shown in Figure 13. From Figure 13, it can be seen that the prediction results based on PLS and SVR show a poor tracking performance. This is because the shallow learning method cannot extract the underlying complex nonlinearity of the data set enough. While the prediction results based on the DVAE and Supervised NDS have a significant improvement in tracking ability in the first 300 samples, it is due to the deep extraction on nonlinearity. However, it can be found that DVAE and Supervised NDS also have a weak prediction performance after about the 300th sample. Instead, the proposed M-CVAE model has an outstanding tracking capacity throughout the process, especially in the last 200 samples with a big change. The reason is that the DCVAE and Supervised NDS model cannot solve the instability and uncontrollability problems in VAE model data generation. This is because, with the expansion of the coding area, the randomness and uncertainty of the sampling will also increase, increasing the uncontrollability and randomness of the generated data. As a result, the similarity between the generated data output by the decoder and the original data is reduced. On the contrary, the M-CVAE model constrains the sampling range of the specified label area by adding the label of the original data as a condition to the model. In this way, the generated data can be closer to the original data, and the generalization ability and robustness of the model can be ensured. Because the labels’ input to the encoder and decoder will change with the input data, the input data is always input to the encoder and decoder together with the matched labels. Therefore, the label always specifies that the generated data are as close as possible to the current input sample. In this way, the M-CVAE shows superior fitting performance, even in a big fluctuation. The prediction error is further shown in Figure 14. The detailed information of MAE, RMSE, and R^2^ are given in Table 6. From Figure 14 and Table 6, it can be seen that the performance of prediction of the proposed M-CVAE model is superior to other methods.

## 4. Conclusions

In this paper, a new M-CVAE model has been proposed for soft sensor application in industrial processes. The proposed M-CVAE model can not only extract nonlinear characteristics effectively, but can also make the predicted output have the superior ability to track the actual value. Through a comparative study of two industrial cases, it was found that when the process variables change greatly, the proposed M-CVAE model can still obtain better prediction performance by adding the label of the original data as a condition to the model to constrain the resampling range of the specific label area.

For further research on soft sensors, the simultaneous extraction of the multiple characteristics underlying the data should be further considered. In this paper, nonlinearity is deeply mined to describe process characteristics. However, in real industrial processes, other data features, such as the dynamic non-Gaussian feature, are all contained in data. If the dynamic feature and non-Gaussian distribution of the latent variables can be further considered, it will be more helpful to improve the accuracy and robustness of the soft sensor model. Another important issue is that the proposed M-CVAE model can be extended to a semi-supervised model to deal with the lack of labeled samples.

## Figures and Tables

**Table 1:** Description of process variables.

Process Variables	Descriptions
variable 1	Top temperature
variable 2	Top pressure
variable 3	Reflux flow
variable 4	Flow to the next process
variable 5	Sixth tray temperature
variable 6	Bottom temperature A
variable 7	Bottom temperature B
y	Butane C4 content in IC5

**Table 2:** Evaluation indicators versus different numbers of latent variables.

N	MAE	RMSE	R^2^
1	0.1223	0.1476	0.3805
2	0.1166	0.1433	0.4162
3	0.1191	0.1445	0.4068
4	0.1195	0.1482	0.3761
5	0.1152	0.1424	0.4238
6	0.1099	0.1347	0.4844
7	0.1159	0.1413	0.4329

**Table 3:** Evaluation indices for SVR, PLS, DVAE, Supervised NDS and M-CVAE.

N	SVR	PLS	DVAE	Supervised NDS	M-CVAE
MAE	0.1565	0.1524	0.1257	0.1257	0.1146
RMSE	0.1815	0.1790	0.1477	0.1477	0.1379
R^2^	0.0636	0.0893	0.3798	0.3798	0.4324

**Table 4:** Description of the variables in CO_2_ absorption column.

Process Variables	Descriptions
variable 1	Process gas pressure entering 05E001
variable 2	05F003 liquid level
variable 3	05E003 outlet lean liquid temperature
variable 4	Lean fluid flow to 05C001
variable 5	Semi-lean fluid flow to 05C001
variable 6	05F003 outlet process temperature degree
variable 7	05C001 process gas inlet and outlet pressure difference
variable 8	05C001 outlet-rich liquid temperature
variable 9	05C001 liquid level
variable 10	06F001 high liquid level alarm value
variable 11	Enter the process gas pressure of unit 06
y	Residual CO_2_ content in process gas

**Table 5:** Evaluation indices of different latent variables.

N	MAE	RMSE	R^2^
1	0.0024	0.0028	0.8410
2	0.0023	0.0028	0.8338
3	0.0022	0.0027	0.8475
4	0.0022	0.0027	0.8475
5	0.0023	0.0026	0.8497
6	0.0022	0.0026	0.8537
7	0.0022	0.0026	0.8548
8	0.0022	0.0026	0.8555
9	0.0022	0.0026	0.8530
10	0.0022	0.0026	0.8559
11	0.0021	0.0026	0.8575

**Table 6:** Evaluation indices for each model.

N	SVR	PLS	DVAE	Supervised NDS	M-CVAE
MAE	0.0057	0.0045	0.0036	0.0036	0.0032
RMSE	0.0062	0.0055	0.0043	0.0042	0.0041
R^2^	0.1644	0.3429	0.6012	0.6237	0.6419

## Data Availability

Data is unavailable due to privacy or ethical restrictions.
